# Understanding the role of qualitative evidence synthesis in health and social care guideline development: perspectives from guideline committee members and technical staff in a leading UK producer of health and social care guidelines

**DOI:** 10.1186/s13643-026-03268-w

**Published:** 2026-07-16

**Authors:** Chris Carmona, Christopher Carroll, Susan Baxter

**Affiliations:** 1 University of Sheffield, Sheffield, UK; 2 Independent, Sheffield, UK

**Keywords:** Qualitative evidence synthesis, Health and social care guidelines, Evidence-based healthcare

## Abstract

This paper explores the perceptions and experiences of guideline committee members and technical staff at the UK’s National Institute for Health and Care Excellence (NICE) regarding the use of qualitative evidence syntheses (QES) in the guideline development process. While QES has the potential to enrich guideline development with insights into service user experiences, implementation contexts, and social determinants of health, its integration into the process remains inconsistent. Drawing on 12 semi-structured interviews conducted with members from clinical, public health, and social care committees, and NICE technical staff, this qualitative study aimed to identify factors influencing the committees’ use of QES in making guideline recommendations. Detailed analysis of the data identified three recurring themes: (1) trust in the credibility and robustness of QES; (2) challenges in the presentation and comprehension of QES; and (3) perceived value and limitations of QES in decision-making. Participants described uncertainty about qualitative methodologies, a reliance on the quantitative hierarchy of evidence, and variability in the quality and clarity of QES reporting. Despite these concerns, many saw QES as vital in amplifying service user voices and supporting lay-member engagement. The findings underscore the need for improved methodological transparency, clearer integration of qualitative and quantitative evidence, and capacity building in QES methods. This study contributes to the literature on evidence-informed policy and practice, highlighting key challenges and offering practical recommendations for enhancing the role of QES in guideline development.

## Background

Guideline-producing organisations like the UK National Institute of Health and Care Excellence (NICE) are central to shaping clinical, public health, and social care practices in the UK and around the world [23]. These guidelines traditionally prioritise quantitative evidence from randomised controlled trials, systematic reviews, and economic analyses. This hierarchy of evidence—favouring experimental over experiential data—has long been critiqued in evidence-based healthcare (EBHC) for under-representing the social, cultural, and contextual dimensions that influence health outcomes [11].

In recent years, qualitative evidence syntheses (QES) have gained recognition as valuable tools to explore patient perspectives, barriers to access, and the implementation context of interventions. QES can inform guideline recommendations by complementing quantitative findings and uncovering issues not amenable to quantification. Despite growing support in the academic literature, and initiatives and utilisation by institutions such as the World Health Organization (WHO) [18, 28, 31], the application of QES in practice remains inconsistent. This is particularly true within NICE, where QES have been described as underused, undervalued, and underreported [9].

Guideline committees at organisations such as NICE are typically composed of multidisciplinary members, including clinicians, public health and social care experts, academics, service users, and technical staff. Their role is to interpret and apply evidence syntheses—both quantitative and qualitative—to formulate recommendations that are evidence-based, implementable, and contextually appropriate [23]. The process is iterative and deliberative, involving structured discussions of the evidence, consideration of its quality and relevance, and reflection on factors such as equity, acceptability, and feasibility. While quantitative evidence often forms the backbone of intervention effectiveness assessments, QES are increasingly recognised for their ability to illuminate patient experiences, implementation barriers, and contextual factors that influence real-world outcomes [16, 25].

The integration of QES into guideline development is supported by internationally applied frameworks such as GRADE-CERQual, which enable committees to assess confidence in qualitative findings using transparent criteria [19]. However, research on evidence-informed decision-making suggests that the uptake of evidence depends not only on methodological quality, but also on perceptions of credibility, legitimacy and relevance among decision-makers [13, 20, 32]. Guideline development is increasingly understood as a deliberative process in which research evidence is interpreted alongside professional expertise, contextual knowledge, values and stakeholder perspectives rather than being applied mechanically [15, 17]. Similarly, a growing literature on patient and public involvement suggests that lay members contribute experiential knowledge that can improve the relevance, acceptability and legitimacy of recommendations and help ensure that guideline processes remain responsive to the needs of people affected by health and social care decisions [2, 5]. Within this broader context, questions about how QES is understood, trusted and used by guideline committees extend beyond methodological considerations alone and engage wider debates about evidence utilisation, credibility and the contribution of lived experience to decision-making. Despite this, relatively little empirical research has examined how committee members and technical staff perceive and use QES during guideline development.

A systematic review conducted by these authors [10] revealed that while there is a methodological consensus on how to conduct QES for guideline development (e.g. ENTREQ, GRADE-CERQual) [19, 30], practical application can vary considerably, including at an organisation like NICE. A subsequent content analysis of NICE QES documents demonstrated inconsistencies in reporting standards, transparency, and clarity—issues likely to affect the credibility and uptake of QES among guideline committees [9].

This paper builds on this previous work by presenting the results of a primary qualitative study exploring the perspectives of NICE technical staff and guideline committee members who have experience with QES. The aim is to better understand how QES are perceived, what supports or hinders their use in guideline production, and what changes could facilitate greater integration of qualitative evidence.

## Methods

### Study design and epistemological framework

A qualitative, exploratory study was conducted using semi-structured interviews and thematic analysis. Critical realism informed the study design by allowing exploration of how personal experience, institutional positioning, and methodological familiarity all might shape the use of QES [1].

### Participants and sampling

A total of 12 participants were recruited using purposive sampling to ensure diverse representation across guideline domains (clinical, public health, and social care), roles (committee members, technical staff), and professional backgrounds (medical, non-medical, lay). All participants had recent experience with guidelines that incorporated QES.

Three guideline committees were identified that had considered QES as part of their evidence base. From each, two to three members and two NICE staff involved in QES preparation or presentation were invited. Participants were approached via email following an in-meeting presentation about the research project. All those who responded to the invitation were able to participate, yielding a total of 12 interviews.

The three committees represented different areas of NICE guideline development, each characterised by somewhat different evidence landscapes. Clinical guideline questions are often informed primarily by randomised controlled trials, whereas public health and social care topics more frequently draw on observational studies and, in some cases, expert testimony where empirical evidence is limited. These differences may shape committee familiarity with different forms of evidence and influence how evidence is discussed during recommendation development. However, the study was not designed to compare committee types and no clear differences between clinical, public health and social care participants emerged from the analysis (Table 1).

**Table 1:** Overview of participants

Committee	Individual*	Committee role	Professional role
Clinical	CM011	Committee member	Non-medical
Clinical	CM012	Chair	Medical
Clinical	CM013	Lay-member	Public
Clinical	TS014	Technical (senior)	NICE staff
Pub health	CM021	Committee member	Medical
Pub health	CM022	Lay-member	Public
Pub health	TS023	Technical	NICE staff
Pub health	TS024	Technical	NICE staff
Social care	CM031	Committee member	Non-medical
Social care	CM032	Committee member	Medical
Social care	TS033	Technical	NICE staff
Social care	TS034	Technical (senior)	NICE staff

### Data collection and topic guide development

Data collection started during the COVID-19 epidemic, and, because of restrictions, interviews were conducted online using Google Meet, with audio recording enabled. Each interview lasted between 45 and 60 min. A topic guide, developed through consultation with co-authors and literature on best practice in qualitative interviewing, covered the following areas that were identified as important from the previous methodological review and content analysis:

- Familiarity with qualitative research and QES
- Perceived value and role of QES in recommendations
- Experiences of QES presentation and interpretation
- Reflections on strengths, weaknesses, and improvements

Participants were provided with the topic guide in advance, along with an information sheet and consent form. All interviews were conducted by the lead researcher, a NICE employee and qualitative researcher.

### Ethics and reflexivity

Ethical approval was granted by the University of Sheffield (Ref: 035876). All participants gave written informed consent. Anonymity was preserved using unique identifiers (e.g. TS034, CM012). Interview recordings were transcribed verbatim and anonymised, with transcripts stored securely.

Given the first author’s employment at NICE, insider status and reflexivity were central considerations. Insider research offers advantages such as contextual access and rapport, but also presents risks including role duality, confirmation bias, and interpretive assumptions [3, 14]. To mitigate these, the primary author maintained a reflexive journal and engaged in peer debriefing with co-authors to surface assumptions and critically examine positionality. These reflections informed data interpretation, helping to identify how professional background, prior beliefs about QES, and interpersonal dynamics may have shaped analysis. Participants were informed that the research was independent of NICE’s formal processes, and efforts were made to minimise power imbalances, particularly with colleagues. While insider status enabled richer insights into organisational practice, reflexive strategies were used to ensure transparency, credibility, and ethical integrity [4, 27].

### Data analysis

Data were analysed using reflexive thematic analysis following Braun and Clarke’s [6] six-phase framework:

1. Familiarisation with the data
2. Generation of initial codes
3. Searching for themes
4. Reviewing themes
5. Defining and naming themes
6. Producing the report

NVivo 12 software [21] was used to support storage and retrieval of textual data. Codes were developed inductively, although sensitised by insights from an earlier systematic review [10] and content analysis [9]. Development of the coding framework was reviewed iteratively with co-authors and refined and revised by reviewing data within each code and analysing coherence across codes and across the framework.

## Findings

**Fig. 1:**
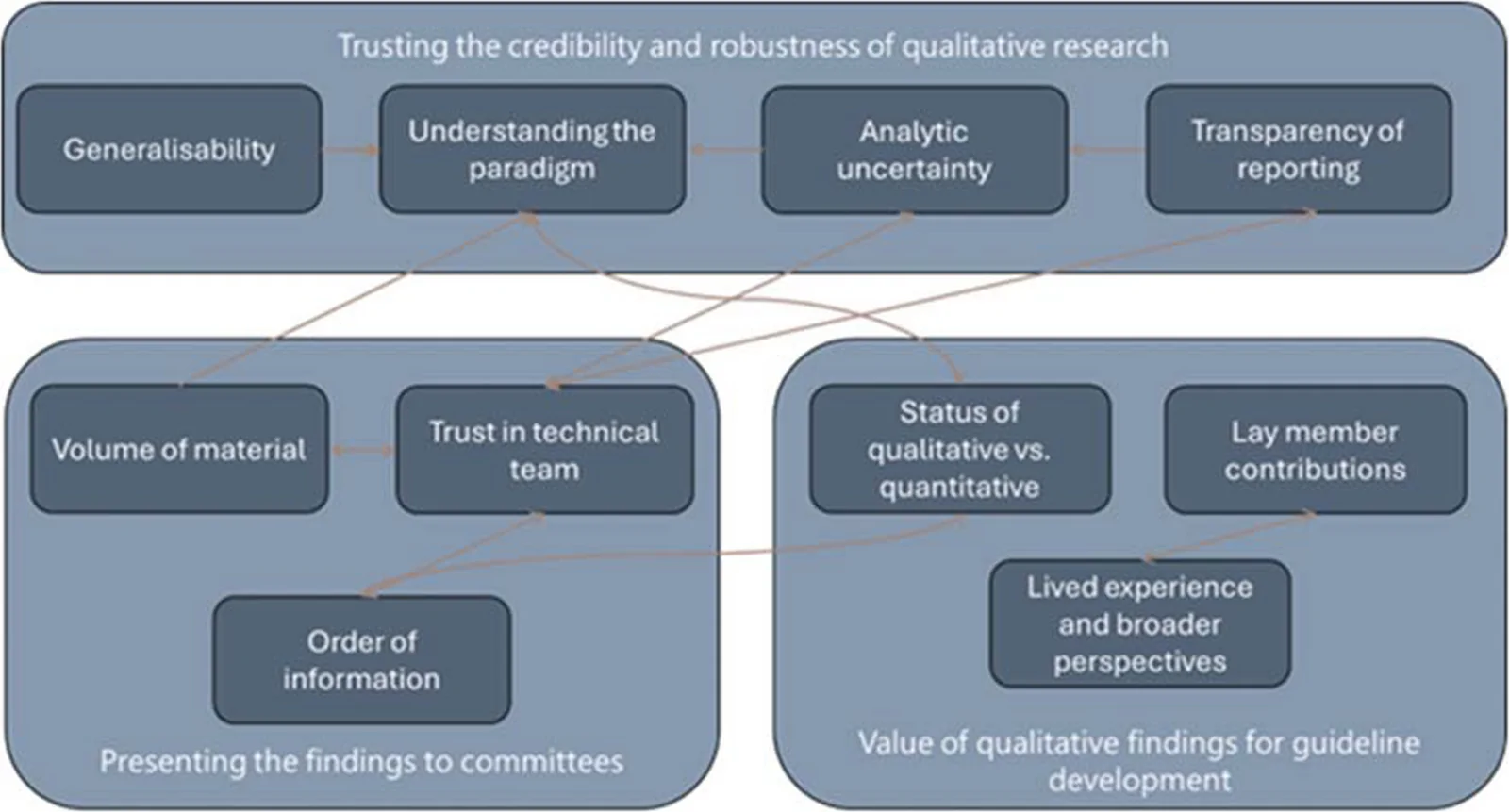
Themes and sub-themes

This section outlines data relating to the three key recurring themes and 10 sub-themes identified during analysis of participant interviews (see Fig. 1), The main themes were: (1) trust in credibility and robustness of QES (4 sub-themes), (2) presentation and interpretation of findings (3 sub-themes), and (3) perceived value of QES in guideline decision-making (3 sub-themes).

Although the thematic framework was presented in three distinct sections, the analysis revealed a complex web of interdependencies between themes and sub-themes (see Fig. 1). Central to these interconnections was the issue of trust in the credibility and robustness of qualitative research (“Theme 1: trust in the credibility and robustness of QES” section), particularly shaped by participants’ understanding of the qualitative paradigm (“Lack of familiarity with qualitative paradigms” section). Limited understanding often led to analytic uncertainty (“Analytic uncertainty” section) and concerns about generalisability (“Generalisability and sample size concerns” section), which in turn increased reliance on the technical team (“Trust in the technical team” section). This reliance prompted the need for a greater volume of material (“Volume of material” section) to ensure transparency and mitigate committee scepticism.

Importantly, these dynamics were not uniformly experienced. Committee members appeared largely unaware of their limited grasp of qualitative methods, whereas technical staff openly acknowledged their lack of training and experience in qualitative synthesis. This asymmetry further influenced how evidence was interpreted and presented, reinforcing the interdependence between epistemological understanding, presentation strategies, and trust in the synthesis process.

### Theme 1: trust in the credibility and robustness of QES

Participants frequently expressed concerns regarding the perceived robustness of QES. These concerns were linked to a lack of familiarity with qualitative methods, leading to discomfort with ideas of transferability and a sense of having to rely more heavily on technical staff. They clustered around 4 key ideas.

#### Lack of familiarity with qualitative paradigms

Several committee members found the epistemological foundations of qualitative research difficult to grasp. The uncertainty seemed to be related to limited understanding of the methods and underpinning principles of qualitative research generally. Most participants extrapolated their understanding of quantitative research within a positivist paradigm to qualitative methods, and from that perspective found it lacking:

I think, feel on confident ground when it comes to reviewing the evidence reports on the quantitative synthesis in that, you know, there’s a nice forest plot there or there’s that the…the numbers that are being presented are, uh, I guess, more objective in a sense. Whereas my experience of the way that our qualitative reviews tend to be presented is that within the kind of tabular data, we get a single quote, for example, that might illustrate a point. (Medical Chair, clinical committee)

This unfamiliarity created a scepticism towards QES findings, particularly when they lacked a clearly articulated synthesis method.

Overall, committee members tended to regard QES, either overtly or by implication, as a secondary or supplementary form of evidence. A member of NICE technical staff summarised this by reflecting that quantitative data were seen as providing the essential evidence, with the qualitative data being almost complementary:

I feel like committees see the quant results as the real meat of the review, and qualitative as just adding the colour and adding “Oh, that’s interesting. That’s, you know, a nice, interesting extra detail, but we make our recommendations on the numbers because that is the proper science”. (Technical staff, public health committee)

#### Generalisability and sample size concerns

Participants frequently applied quantitative standards to judge QES, especially with regard to ‘generalisability’ (in the quantitative sense) rather than assessing the depth, insights, and contextual detail of the evidence to support transferability [26]:

You know, how can we extrapolate from a you know, it’s relatively small and it comes down to numbers again, is a relatively small population of the, you know, a series of focus group interviews or a series of semi-structured interviews in one population, in one [venue type] in London to extrapolate that out… (Medical committee member, social care committee)

This led several participants to think QES might only be useful as ‘supporting’ evidence, and that committee opinion was needed to gauge the credibility of QES findings:

And of course, the limitation of qualitative data is always, you know, how representative it is. So, I think what’s important is that it’s a combination of quantitative, qualitative, and expert opinion from the committee in this particular context that I think is most valuable. (Non-medical committee member, clinical committee)

Concerns were also raised about the generalisability of findings from international studies to the UK context. One committee member questioned the applicability of evidence from studies conducted in different cultural and socioeconomic settings:

they might do a study in a... poorer ethnic area, but... their societies not identical to ours... would that work with our populations? (Medical committee member, public health committee).

This reflects broader debates within qualitative research about the limits of generalisability and the contextual nature of findings.

#### Analytic uncertainty

Participants reported that it was difficult to understand the analysis process in QES. It was often difficult to understand how the themes reported in the primary studies mapped onto the themes reported in the QES, and this was often not possible to track meaningfully through the QES reporting. They noted that they could quickly match results from quantitative systematic reviews with the original papers (if they had access to the journal), but that this was much more difficult with qualitative papers, not least because they are often longer and lack tables and figures that make checking easy:

…colleagues sat around the table will look at the study that’s being presented and reappraise it for themselves. [Int: Yeah], in order to...to have great confidence and they can do that real time very quickly with the quantitative work. And I think it’s nigh on impossible to do that real time and quickly with the qualitative work, because I need to… I need to read a 20-page paper. [Int: Yeah]. As opposed as opposed to skim read a six-page paper (Medical Chair, clinical committee)

#### Transparency of reporting

Participants highlighted the importance of transparency in systematic reviews and in QES and agreed that QES could be harder for a reader to make sense of than quantitative systematic reviews. They were ‘more opaque and wordy’, and lacked simple tables and figures that they associated with quantitative systematic reviews:

I think there’s you know, there’ll be a summary table, for example, of a description of, you know, that a study with focus groups and questionnaires in a certain set of [settings] in London boroughs. [Int: Yeah], but that’s separate in a sense to the table that presents the findings, which is separate again to the table, to the diagram that might try and pull that together in terms of its… its synthesis. And so it takes work to… to assimilate all of that information. And …and I guess one of the, and I think probably takes more work than it does for the way that the quantitative evidence is presented. So, I think that’s probably one of the challenges. (Medical committee member, social care committee)

This was compounded in larger QES with many themes (see the “Volume of material” section).

### Theme 2: presentation and comprehension of QES findings

Participants described significant variation in how QES findings were presented and how accessible they were. This is grouped into 3 subthemes. 

#### Volume of material

Committee members and technical staff both grappled with the challenges posed by the extensive size of QES, particularly the volume of themes and supporting quotes. The analytic ambiguity and difficulty in tracing themes transparently back to primary studies necessitated a higher level of detail for committee members to feel confident in the synthesis, which inevitably then meant the provision of more materials. Technical staff acknowledged this complexity, noting the difficulty of condensing large QES into presentations that were sufficiently detailed yet not overwhelming.

it’s very easy to just present a forest plot... fairly straightforward to get them to interpret. But... I had to hone the presenting for qualitative...It was a big review [and] everyone just kind of wanted to give up and go home... And so yeah, I know they didn’t engage very much with that at all (Technical staff, public health committee).

Technical staff agreed that the volume of material and papers that they had to synthesise could be prohibitive and they had experienced not only how this could overwhelm committees but were unsure what a solution could be:

But whether we needed something that then rather than presenting all the same themes forever, you know, again, and again, and again, maybe we needed a higher-level integration that was like, these are the themes that have come up for everybody. And then these are the separate themes. (Senior technical staff, clinical committee).

A senior technical staff member described similar issues with a junior colleague:

I had to try and stop him from just putting them [themes and quotes] up and reading them word for word... just like try and summarise them a little bit... Because he did just want to read them straight out… it was just boring as hell (Senior technical staff, social care committee).

Additionally, technical staff emphasised the need for deep familiarity with qualitative content to effectively respond to committee queries, contrasting this with the relative ease of summarising quantitative data:

Yeah, it’s... quite a lot of background knowledge to be able to answer questions on it. Whereas quantitative... everything you need is usually on the slide (Technical staff, public health committee).

#### Trust in the technical team

Participants highlighted the need for increased trust in the technical team when working with QES due to analytical uncertainty, the perceived lack of transparency and the resulting difficulty in independently verifying the synthesis process. This reliance placed greater responsibility on technical staff to present qualitative findings in a manner that reassured committees of the findings’ rigour and robustness.

As one committee Chair observed,

the committee, I think, has to put their trust in the technical teams, possibly even more than they need to with the quantitative work (Medical committee Chair, clinical committee).

While some participants expressed confidence in the technical staff’s capabilities:

Yeah, I’ve always found the technical analyst, really, really good, really clear... really helpful to see how the different studies relate to one another (Lay-member, clinical committee)

Such comments often reflected broader concerns about the opacity of QES methods. Unlike quantitative techniques such as meta-analysis, which are rarely questioned despite the details of the intrinsic statistical methods likely being unfamiliar to many committee members, QES was seen as more methodologically uncertain. This analytic uncertainty led to concerns about the ‘black box’ nature of QES production, where much of the process was seen to rely on the tacit knowledge and interpretive skill of the technical team, particularly given the volume of data involved (see also the “Analytic uncertainty” and “Volume of material” sections).

#### Ordering and framing of evidence presentation

Technical staff reflected on how the sequencing of qualitative and quantitative evidence during committee meetings could influence interpretation and engagement. One participant described presenting qualitative findings first to foreground barriers and contextual challenges, followed by quantitative data as responses to those issues. They perceived that this approach shifted the committee’s perspective:

…I got them thinking about the barriers first… seeing the quantitative in terms of the qualitative rather than the other way around (Technical staff, public health committee).

Although committee members did not explicitly comment on this strategy, several implied that qualitative data often served simply to support or fill gaps in quantitative findings. A technical staff member recalled a typical example of how a committee would make recommendations informed by both types of evidence, but focusing first on the quantitative, and with the final framing still emphasising the primacy of quantitative data:

they made the recs based on the quantitative and also then took the qualitative into account (Senior technical staff, clinical committee).

### Theme 3: value of qualitative findings for guideline development

This theme addressed how participants positioned QES relative to other forms of evidence. Three subthemes were identified within this theme.

#### ‘Supplementary’ status of QES

Some participants expressed openness to qualitative evidence and recognised it to have value:

even what I would describe as the hard-core quantitative people... have found the qualitative evidence important... I felt that the committee can really kind of relate to what’s coming out in the qualitative evidence (Non-medical committee member, clinical committee).

However, the same participant later expressed surprise that NICE considered qualitative evidence sufficient for making recommendations, suggesting its value was conditional: “it becomes very important if that’s all you’ve got for a particular area”. None explicitly recognised scenarios in which qualitative findings might be more central to decision-making than quantitative data; it was typically framed as supplementary.

This perception was echoed by others, who implied that qualitative data served primarily to enrich or contextualise quantitative findings. One technical staff member described it as

the quantitative data was the [clothes] hanger, and the qualitative data was the clothing draping over the hanger (Senior technical staff, clinical committee).

Even in committees familiar with qualitative methods, its usefulness was often linked to the absence of quantitative evidence:

one of the reasons that qualitative evidence was useful, was because there was so little quantitative evidence (Technical staff, social care committee).

Some participants acknowledged that QES could support consensus recommendations, though not necessarily serve as their foundation:

they did use it to bolster what they believe to be true anyway and make a recommendation based on that (Senior technical staff, social care committee)

Nonetheless, technical staff recognised the strategic value of QES in strengthening recommendations during the quality assurance process:

it’s not really showing us anything new... But it’s given us the opportunity to say this is a problem... I’m hoping... there’s less of that fight for that recommendation to stay in (Senior technical staff, social care committee).

Some participants questioned the added value of QES when lived experience was already present within the committee. One Chair suggested that the presence of appropriate lay-members might render QES redundant:

you’d be pretty stupid not to listen to what they [lay-members] have to say... Whether the qualitative research synthesis goes above what those lived experience people will tell you anyway, I’m not convinced (Medical Chair, clinical committee).

This raises important questions regarding the status of personal experiences and views of committee members versus research evidence within the process of guideline development examining both quantitative and qualitative reviews.

#### Lived experience and broader perspectives

Committee members and technical staff consistently identified a key challenge in guideline development: understanding the impact of recommendations on diverse populations, particularly those facing barriers to care. QES was seen as valuable in addressing this gap, given its methodological emphasis on capturing a broad range of perspectives, including those from harder-to-reach groups. Participants noted that while committee members often drew on their own clinical experience of diverse populations, having such insights reflected in QES added credibility and weight to their views.

As one technical staff member explained:

with quantitative evidence... it’s very rarely that the equalities... groups are... covered. But... there was a bit where... one of the papers... said [that] different ethnicities... consider [topic] differently... it’s a given, but it’s nice to have it written down and... documented (Technical staff, public health committee).

This resonance of QES findings with their lived experience, which was interpreted both positively—as validation—and negatively, as the reiteration of the obvious. For example, one technical staff member noted:

it kind of gave them the reassurance... this isn’t just us... it’s in the data as well... this is a massive issue (Technical staff, public health committee).

While a committee member remarked that it simply referred to issues the committee already knew about:

the trouble is, it often ended up being a theme which was perhaps kind of understood anyway, … (Medical committee member, social care committee).

#### QES as enabler of engaging with lay-members

Non-lay participants observed that QES themes could help lay-members validate their own lived experiences, thereby enhancing their confidence and participation in committee discussions. As one committee member noted:

people... have undoubtedly felt reinforced in some of their views and their experiences by hearing... that sort of thing come from other people (Medical committee member, public health committee).

This validation was seen to empower lay-members to contribute more actively, particularly when qualitative evidence aligned with their perspectives:

they were really vocal at that meeting... they all did step up and they all contributed (Technical staff, social care committee).

Committee members also suggested that lay-members engaged more readily with qualitative evidence due to its accessibility and relevance to lived experience, in contrast to the technical nature of quantitative data:

the qualitative evidence gave the lay-members a voice... we’ve looked at very much technical pieces of information where they can’t really chip in (Medical committee member, social care committee).

Similarly, a lay-member speculated:

lay-members would gravitate more towards the qualitative because they lived experience... scientists and medics might pay more attention to the quantitative (Lay-member, clinical committee).

Lay-members themselves expressed appreciation for the broader perspectives offered by QES, noting that it helped them consider experiences beyond their own:

it gives me a lot more to think about when it’s taught me about other people’s experiences... compared to just more numerical values (Lay-member, clinical committee).

This suggests that effective use of QES may expand the scope of lay-member contributions during guideline development.

## Discussion and conclusions

This study provides new insights into the perceptions of guideline developers (technical staff and committee members) at one UK institution about the use of QES in evidence-informed decision-making. Across the interviews in this study, participants consistently acknowledged the potential value of QES in reflecting lived experience and addressing gaps left by quantitative research. Despite this, and the increased institutional support and a growing methodological literature around QES, our findings reveal persistent epistemological and operational barriers that limit its integration and influence.

### Revisiting the hierarchy of evidence

Participants frequently described QES as supplementary to quantitative evidence. In some circumstances this may be entirely appropriate. For example, for questions relating to intervention effectiveness or safety, quantitative evidence will often provide the principal basis for recommendations. However, many guideline questions concern issues such as acceptability, feasibility, implementation, access, equity and lived experience, for which qualitative evidence may provide uniquely relevant insights. The findings suggest that committee members and technical staff did not always distinguish between these different evidentiary purposes, and that QES was often positioned as supplementary by default rather than in relation to the specific decision being addressed. Guideline-producing organisations may therefore benefit from clearer guidance on the different roles that qualitative evidence can play in recommendation development, including when it is primarily contextual, when it complements effectiveness evidence, and when it may directly inform recommendations addressing experience, implementation, or service delivery.

Participants’ tendency to assess QES using quantitative criteria, such as sample size and generalisability, also suggests a broader limited understanding of qualitative paradigms. This is consistent with previous work showing that non-specialists often struggle to appraise qualitative research due to its perceived subjectivity and lack of standardised outputs [12].

### Credibility and trust in qualitative findings

A major finding of this study is that trust in QES often rests more on the perceived credibility of the technical team than on the evidence itself. This reliance on interpersonal trust rather than methodological understanding raises concerns about the sustainability and scalability of QES integration in guideline production. While expert facilitation is crucial, it must be accompanied by broader institutional capacity building to ensure committees can appraise and engage with QES independently.

### Challenges in presentation and interpretation

Participants identified a lack of visual clarity and large volumes of findings as significant barriers to QES engagement. These issues are well documented in the literature [25, 29] and point to the need for better synthesis reporting tools. Structured, layered formats that provide concise summaries alongside richer contextual data could help committees to engage more fully and effectively.

The influence of verbal presentation during meetings further underscores the importance of communication style in evidence uptake. This finding suggests that investing in training for technical staff on how to present QES concisely and compellingly can be as important as methodological rigour.

The findings also reveal an important tension in the reporting and presentation of QES. Participants frequently described a desire for greater transparency, richer explanations of analytic decisions, and clearer links between synthesis findings and the underlying primary studies. However, these same participants also identified the volume and complexity of qualitative evidence reports as a barrier to engagement. This creates a practical dilemma: increasing methodological detail may improve confidence and transparency but may simultaneously increase cognitive burden and reduce accessibility for committee members. Conversely, highly condensed summaries may improve usability but risk stripping findings of the contextual detail that gives them meaning and may reinforce perceptions that qualitative findings are general, predictable, or self-evident. The challenge for guideline developers is therefore not simply to provide more information or less information, but to identify reporting and presentation approaches that balance transparency with usability.

### Value and limitations of QES in NICE guidelines

QES was widely acknowledged to offer benefits in legitimising lay-member perspectives and addressing issues overlooked by quantitative research. However, its perceived utility remained limited to corroborating rather than generating recommendations. This aligns with studies that suggest qualitative evidence is often used instrumentally—to support decisions already made—rather than constitutively, as a driver of new insights [8].

Some participants suggested that the presence of lay-members with lived experience might reduce the need for QES. However, this view reflects a potentially concerning perception that the perspectives of one or two individuals can substitute for the breadth, representativeness, and rigour of a formal synthesis. QES typically draws on diverse populations across multiple studies and contexts, offering systematically derived insights that go beyond individual testimony.

### Strengths and limitations

A key strength of this study is its insider perspective, which allowed for nuanced understanding of NICE’s internal processes and cultures. The researcher’s insider status, while enabling access and depth, may also have introduced bias, despite robust reflexivity measures. This can be related, for example, to role duality, taking organisational norms for granted, or interpreting data in ways that confirm pre-existing beliefs [7, 22, 24, 33]. This was mitigated by extensive discussion of emerging themes with co-authors who are not connected with NICE.

The sample, though diverse in role and domain, was relatively small and drawn from only three guideline committees. Finally, the findings are specific to one organisation in the UK and may not fully generalise to other guideline-producing contexts.

Although the sample included clinical, public health and social care committees, the study was not designed to undertake systematic comparisons between these groups. It is plausible that committees working in areas with greater reliance on observational, qualitative or experiential evidence may develop different approaches to evidence appraisal and deliberation. However, participants across all three domains described broadly similar challenges relating to trust, credibility, and interpretation of QES.

### Recommendations for policy and practice

Based on the findings, we propose the following recommendations:

1. Training and capacity building: introduce regular training for both committee members and technical staff on qualitative methods and QES interpretation. Include workshops, case studies, and interactive tools.
2. Enhanced reporting standards: maintain adherence to established QES reporting standards (e.g. ENTREQ, GRADE-CERQual) while exploring layered reporting approaches that allow committees to access differing levels of methodological and contextual detail according to their needs.
3. Integrated presentation formats: explore presentation formats that support rapid understanding whilst preserving access to contextual detail, such as mixed-methods matrices, layered summaries, visual evidence displays, and linked evidence tables.
4. Clarify role of QES in recommendations: develop internal guidance on when QES can directly inform recommendations and provide examples from past guidelines to illustrate best practice.
5. Support evidence literacy across different forms of evidence: training and committee development activities should focus on helping committee members understand the distinct purposes, strengths, and limitations of different forms of evidence, including quantitative, qualitative, and experiential evidence. This may help reduce inappropriate application of quantitative criteria to qualitative findings and support more evidence informed deliberation**.**

## Conclusion

This study highlights the potential of QES to enhance guideline development, but also reveals the epistemic, communicative, and structural barriers to its effective use currently. By investing in training, improving reporting and presentation, and revisiting institutional assumptions about evidence, guideline producers can potentially more fully realise the benefits of qualitative synthesis.

Further research should explore the implementation of these recommendations and examine the impact of enhanced QES integration on the quality and inclusivity of guideline recommendations.

## Data Availability

This study generated qualitative evidence in the form of interview transcripts and field notes. These materials contain identifiable information and cannot be made publicly available. Enquiries about access may be directed to the corresponding author.
